# Selective Formation of Twisted Intramolecular Charge Transfer and Excimer Emissions on 2,7-bis(4-Diethylaminophenyl)-fluorenone by Choice of Solvent

**DOI:** 10.3390/molecules17044452

**Published:** 2012-04-13

**Authors:** Masayuki Shigeta, Mifumi Morita, Gen-ichi Konishi

**Affiliations:** 1 Department of Organic and Polymeric Materials, Graduate School of Science and Engineering, Tokyo Institute of Technology, Tokyo 152-8552, Japan; 2 PRESTO, Japan Science and Technology Agency, Saitama 332-0012, Japan

**Keywords:** twisted intramolecular charge transfer (TICT), excimer, intermolecular charge transfer, fluorenone, donor acceptor dye

## Abstract

We designed and synthesized a donor-acceptor-donor dye consisting of a 2,7-disubstituted fluorenone with diethylaminophenyl moieties present as strong electron donating groups. Switching between twisted intramolecular charge transfer (TICT) emission and excimer emission was achieved, with no ground state changes, by simply changing the solvent used. In a nonpolar solvent, excimer emission was observed; with increasing polarity, the emission gradually disappeared, and the TICT emission appeared.

## 1. Introduction

Twisted intramolecular charge transfer (TICT) with donor-acceptor dyes is a basic phenomenon utilized in various applications, and investigations have been carried out in order to analyze this phenomenon in detail [1,2]. One of the aims of these studies was to achieve TICT emission with the least possible participation of other relaxation mechanisms, such as emission from locally excited (LE) states and interactions within a local state. The criteria for this purpose were determined on the basis of extensive investigations; these criteria specify rigorous dye design [3,4] in addition to control parameters, such as solvent choice and temperature [5,6,7,8,9]. Consequently, this phenomenon can be applied to dye-sensitized solar cells [10,11,12], OLEDs [13,14], and chemosensors [15,16,17].

**Scheme 1:**
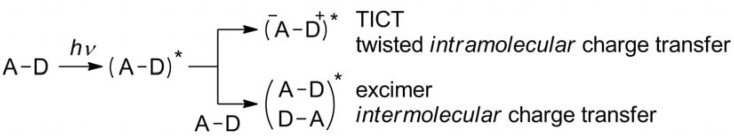
TICT and excimer formation by a rigid dye consisting of a donor and an acceptor [18,19].

In the case when a donor-acceptor dye forms an excimer, the ratio of TICT emission decreases, and excimer emission appears (Scheme 1) [18,19]. This had raised the possibility that switching between TICT and excimer emissions could be easily facilitated by environmental changes in the surroundings. Subsequently, such switching was achieved by changes in concentration [20] or aggregation stimuli [21]. However, to date, there has been little investigation on the effect of changing the solvent used, which is a very simple environment change [19]. Previous studies have reported only shifts of the emission peaks when the solvent was changed [22,23,24].

In this paper, we report a newly synthesized donor-acceptor type dye that can be switched between TICT and excimer emission, without any ground state changes, by simply changing the solvent. We hypothesized that fluorenone was a suitable structure to use in this study owing to its tendency to form excimers [25]. Furthermore, the photophysical properties of fluorenone derivatives have been studied in detail including their dependence on various changes in the surrounding environment [26,27,28,29,30,31,32,33]. Various fluorenone derivatives have been synthesized with an attached amino moiety as a strong electron donor group. The direct attachment of this donor group to the fluorenone molecule changes the spectra in the ground state [34], and the addition of a rigid π-spacer, such as a phenylene or ethynylene group, inhibits the ground state interaction [22]. These spacer-containing compounds were also shown to display TICT emission behavior [35]; however, they have not been sufficiently investigated.

## 2. Results and Discussion

**Scheme 2:**
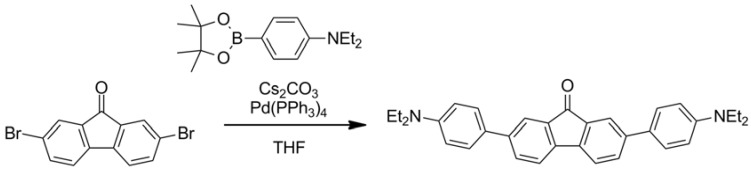
D-A-D type fluorenone dye synthesis.

The structure of the dye and its synthesis condition are shown in Scheme 2. The dye is based on fluorenone, with two diethylaniline moieties attached at the 2,7-positions. The dialkylamine moiety is a very strong electron donating species, and the phenylene moiety is used as a spacer between the donor and acceptor parts to control the photophysical properties of the molecule [22,35].

The dye was synthesized via a Suzuki-Miyaura coupling [36] using 2,7-dibromofluorenone and 4-diethylaminophenylboronic acid pinacol ester over Pd(PPh_3_)_4_ and Cs_2_CO_3_ catalysts in THF at 60 °C for 2 days.

**Figure 1:**
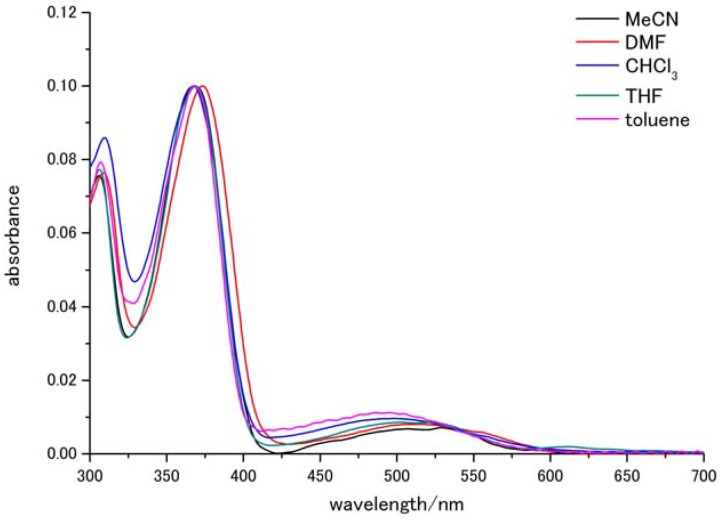
Absorption spectra in various solvents (smoothed by an FFT filter).

We obtained absorption spectra of the compound in various solvents: acetonitrile, DMF, chloroform, THF, and toluene. The dye concentration was set as the concentration at which a maximum absorbance of 0.1 was achieved. Figure 1 shows the collected spectra, which have been smoothed by an FFT filter to reduce the noise due to vibration. The spectra consist of three absorption peaks, which are detailed in Table 1. The wavelengths of the peaks do not deviate much from 310, 370, and 520 nm, indicating that solvent changes do not cause any change in the ground state. The two high-energy peaks were due to fluorenone and were assigned to n-π* or π-π* absorptions, whereas the low-energy absorption peak, at around 500 nm, was due to charge transfer assigned with reference to the previous investigation [22]. However, it is difficult to determine whether the peak is n-π* or π-π*, because only slight solvatochromic effects were observed.

**Figure 2:**
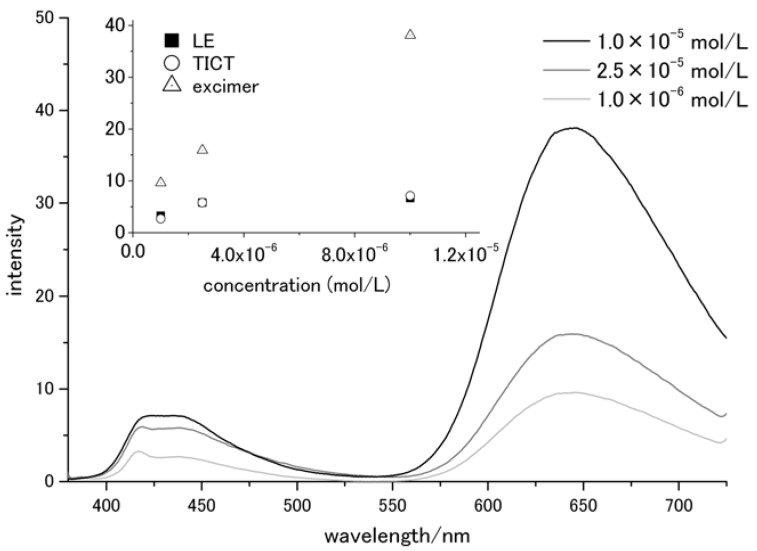
Concentration dependence of fluorescence spectra in THF at 370 nm excitation.

**Figure 3:**
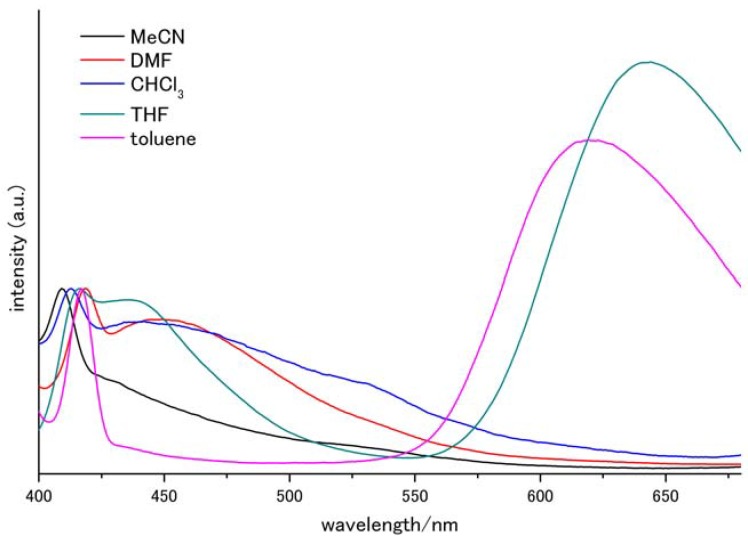
Fluorescence spectra in various solvents.

The concentration dependence of the fluorescence properties was examined in THF. The spectra were measured at 370 nm excitation in order to prevent the irradiation of charge-transferred species. The obtained fluorescence spectra are shown in Figure 2. Three peaks were detected: one sharp peak at 416 nm and two broad, structureless peaks at approximately 436 nm and 644 nm. The fluorescence intensity of the peak at 644 nm increased constantly with dye concentration from 1.0 × 10^−6^ to 1.0 × 10^−5^ mol/L. For the other two peaks, the intensities gradually stopped increasing. This suggested that the 645 nm fluorescence peak was due to excimer emission, and the other two peaks originated from single molecule emissions. From previous reports [19,22], and the solvatochromic effects shown in Figure 3, it was deduced that the sharp peak at 417 nm was due to emission from the LE state and the broad peak at around 435 nm was due to the TICT state. Their wavelengths are summarized in Table 1. It was concluded that these peaks were not due to Raman scattering, because their intensity is higher than that of scattered excitation light and also because a Raman peak was observed at around 390 nm (not shown).

We then investigated the effects of different solvents on the emission spectra, and the obtained spectra are shown in Figure 3, with the heights normalized to the LE peak, the existence of which was independent of the tested solvents. Drastic changes were observed between TICT and excimer emission. Toluene enhanced the excimer emission (λ_em_ = 619 nm). In more polar solvents (that is, chloroform, DMF, and acetonitrile), TICT emission was observed and no excimer emission was detected.

The absorption peaks, the emission peaks, and their normalized solvent polarity parameters are summarized in Table 1. Excimer emission was observed in toluene; with increasing solvent polarity, the excimer peak red-shifted until it disappeared, and the TICT peak appeared. The TICT peak also exhibited positive solvatochromic behavior.

TICT formation was expected to be faster than excimer formation, as it is an intramolecular event. Therefore, in all polar solvents, the broad emission was found to be due to TICT [24]. In the less-polar solvents, toluene and THF, excimer emission was observed, and it is hypothesized that either a TICT was not formed or once a TICT was formed, it was converted into an excimer by a Coulombic force, such as the Harpooning effect [37]. Under this hypothesis, it is possible that TICT stability was enhanced by polar solvents, resulting in the absence of excimer emission.

**Table 1:** Photophysical parameters of the fluorenone dye.

Solvent (line color)	λ_abs_/nm	λ_em_/nm			*E*^N^_T_^b^
		LE	TICT	excimer	
MeCN (―)	307, 369, 529	409	- ^a^	-	0.460
DMF ( 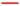 )	310, 372, 520	419	450	-	0.386
CHCl_3_ ( 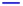 )	310, 366, 503	413	442	-	0.259
THF ( 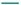 )	307, 371, 522	416	436	644	0.207
toluene ( 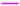 )	308, 371, 496	417	-	619	0.099

^a^ The peak wavelength could not be measured, but broad emission was observed; ^b^ Normalized solvent polarity parameters were cited from [38].

## 3. Experimental

### 3.1. Instruments

All the ^1^H- and ^13^C-NMR spectra were recorded on a 400 MHz JEOL LMN-EX400 instrument with tetramethylsilane (TMS) as the internal standard. FT-IR spectra were recorded on a JASCO FT-IR 469 plus spectrometer. Melting points were obtained by a Stuart Scientific Melting Point Apparatus SMP3. MS spectra (FAB+) were obtained by JEOL JMS700 mass spectrometer. UV-vis spectra were recorded by a Beckman Coulter DU800 UV-vis Spectrophotometer. Fluorescence spectra were recorded by a JASCO FP-6500 Spectrofluorometer.

### 3.2. Materials: Synthesis of 2,7-(4-Diethylaminophenyl)-fluorenone

To a mixture of 2,7-dibromofluorenone (0.10 g, 0.30 mmol), Pd(PPh_3_)_4_ (0.017 g, 0.0015 mmol), Cs_2_CO_3_ (0.68 g, 2.1 mmol), in THF (20 mL) was added 4-diethylaminophenylboronic acid pinacol ester (0.18 g, 0.65 mmol), and the mixture was stirred at 60 °C. After stirring 2 days, the mixture was cooled and filtrated with CH_2_Cl_2_ to remove insoluble solids. Then, the filtrate was extracted with CH_2_Cl_2_ washed with brine, and dried over MgSO_4_. After removal of solvent *in vacuo*, the residue was purified by silica gel column chromatography (hexane/CH_2_Cl_2_ = 1/1.5) and recrystallization to afford the title compound as a purple solid (0.087 g, 0.18 mmol, 61%). MP 274–276 °C; ^1^H-NMR (CDCl_3_): δ 1.21 (t, 12H, *J* = 7.3 Hz), 3.41 (q, 8H, *J* = 7.3 Hz),6.75 (d, 4H, *J* = 8.8 Hz), 7.50 (d, 2H, *J* = 9.5 Hz), 7.52 (d, 4H, *J* = 8.8 Hz), 7.66 (dd, 2H, *J* = 1.7, 9.5 Hz), 7.87 (d, 2H, *J* = 1.7 Hz); ^13^C-NMR (CDCl_3_): δ 12.7, 44.4, 112.1, 120.3, 121.8, 126.7, 127.6, 131.7, 135.3, 141.95, 141.96, 147.7, 194.5; IR (KBr): ν_max_ 3,438, 2,972, 2,929, 2,884, 2,193, 1,720, 1,610, 1,600, 1,518, 1,464, 1,399, 1,376, 1,358, 1,268, 1,198, 1,184, 1,156, 1,127, 1,079, 1,012, 911, 833, 815, 786, 773 cm^−1^; HRMS (FAB+) [M+Na]^+^ Calcd for C_33_H_34_N_2_ONa: 497.2564, Found : 497.2555.

## 4. Conclusions

In this work, we designed a donor-acceptor-donor dye based on fluorenone, in order to achieve switching between TICT and excimer emission without ground state changes, by the simple expedient of changing the solvent. The dye was synthesized via a Suzuki-Miyaura coupling, and absorption and emission spectra in various solutions were obtained. The results indicate that excimer formation was enhanced in nonpolar solvents and that by increasing the solvent polarity, the excimer quantum yield decreased gradually and a TICT peak appeared. This unique phenomenon constitutes a very promising basis for the development of solvent polarity sensors.

## Supplementary Materials

Supplementary materials can be accessed at: http://www.mdpi.com/1420-3049/17/4/4452/s1.
