# Supplementary material for: Selective Formation of Twisted Intramolecular Charge Transfer and Excimer Emissions on 2,7-bis(4-Diethylaminophenyl)-fluorenone by Choice of Solvent
Source: Molecules. 2012 Apr 13;17(4):4452–9. doi: 10.3390/molecules17044452 (PMC6268939; doi:10.3390/molecules17044452)

2,7-(4-Diethylaminophenyl)-fluorenone

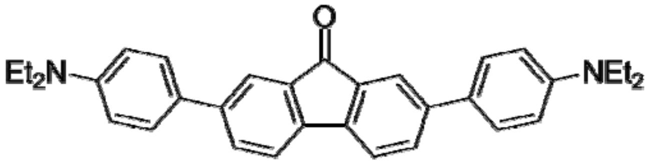

Figure S1. Absorption spectra in various solvents (without smoothing).

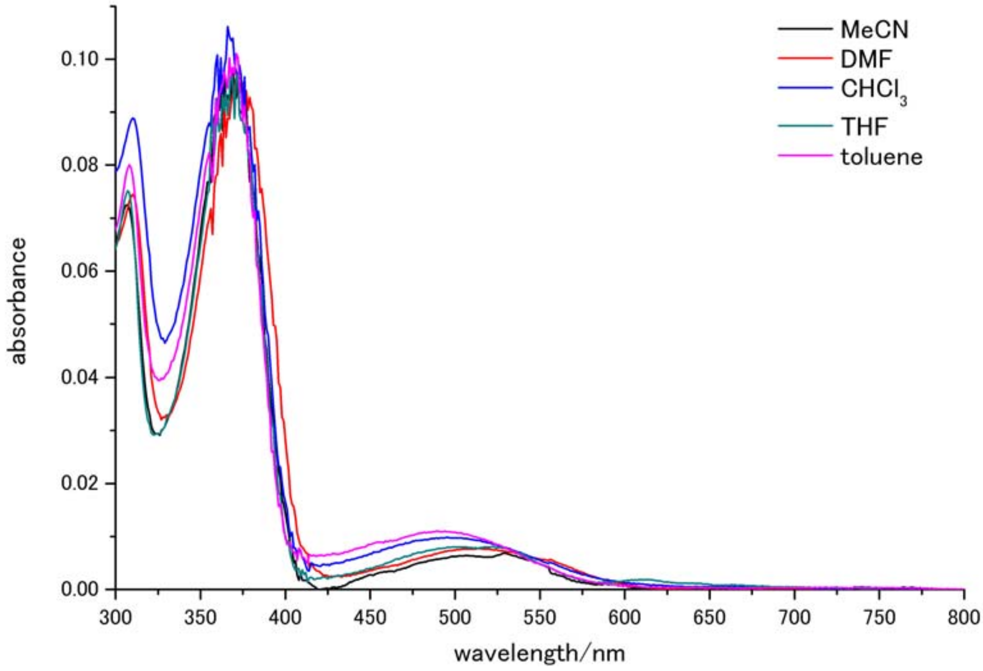

Figure S2. IR.

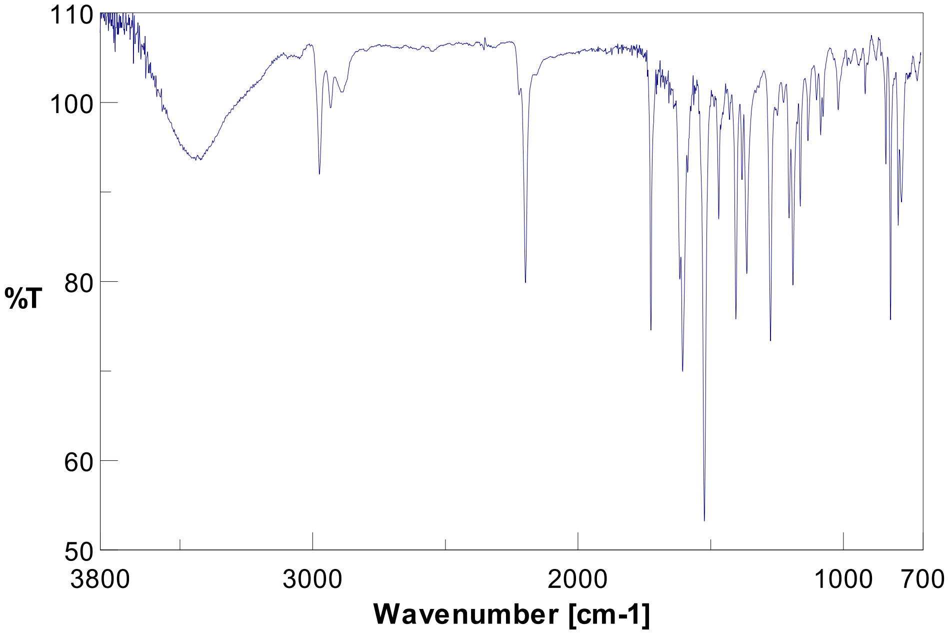

Figure S3. ^{1}H-NMR.

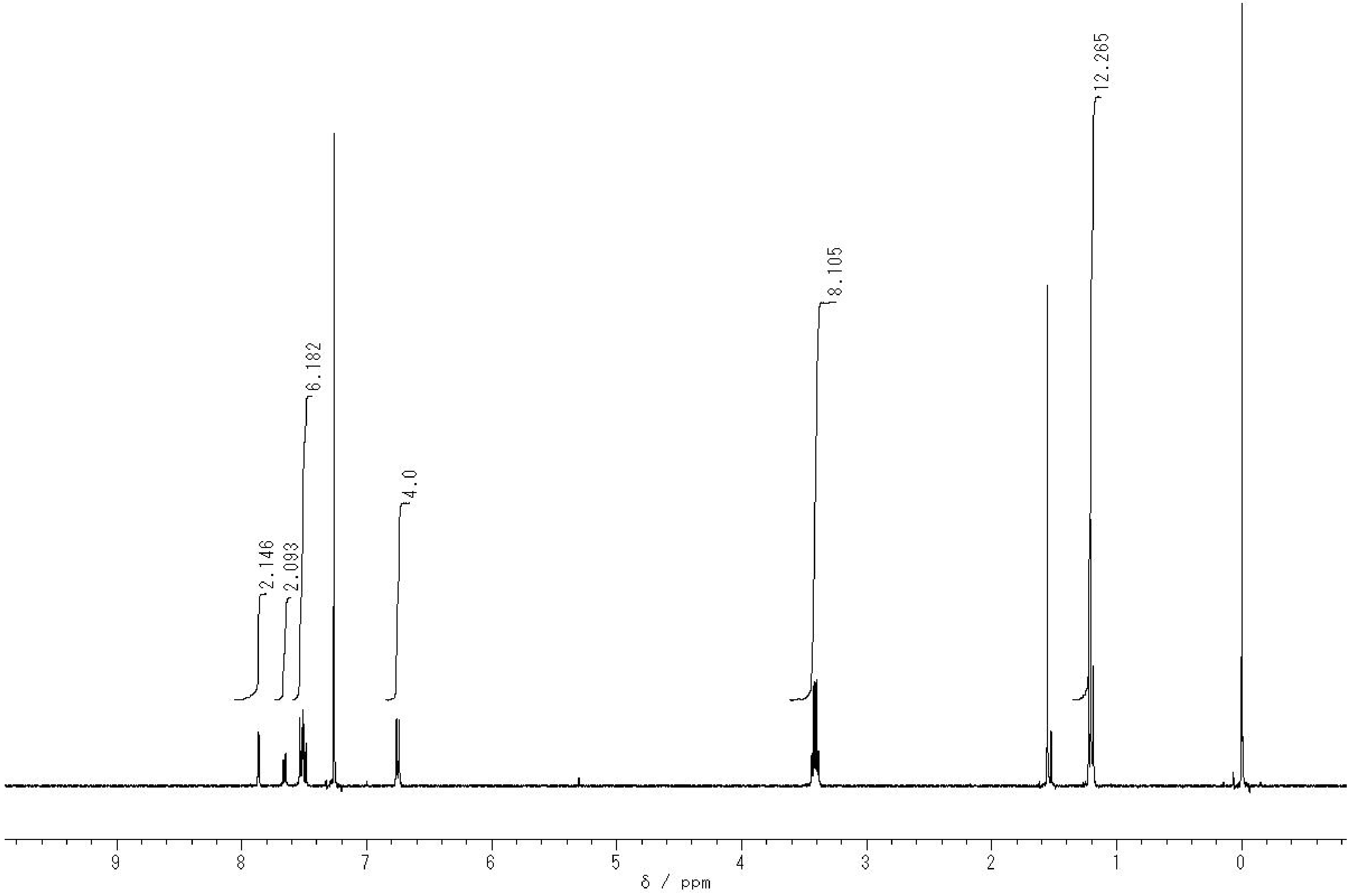

Figure S4. ^{13}C-NMR.

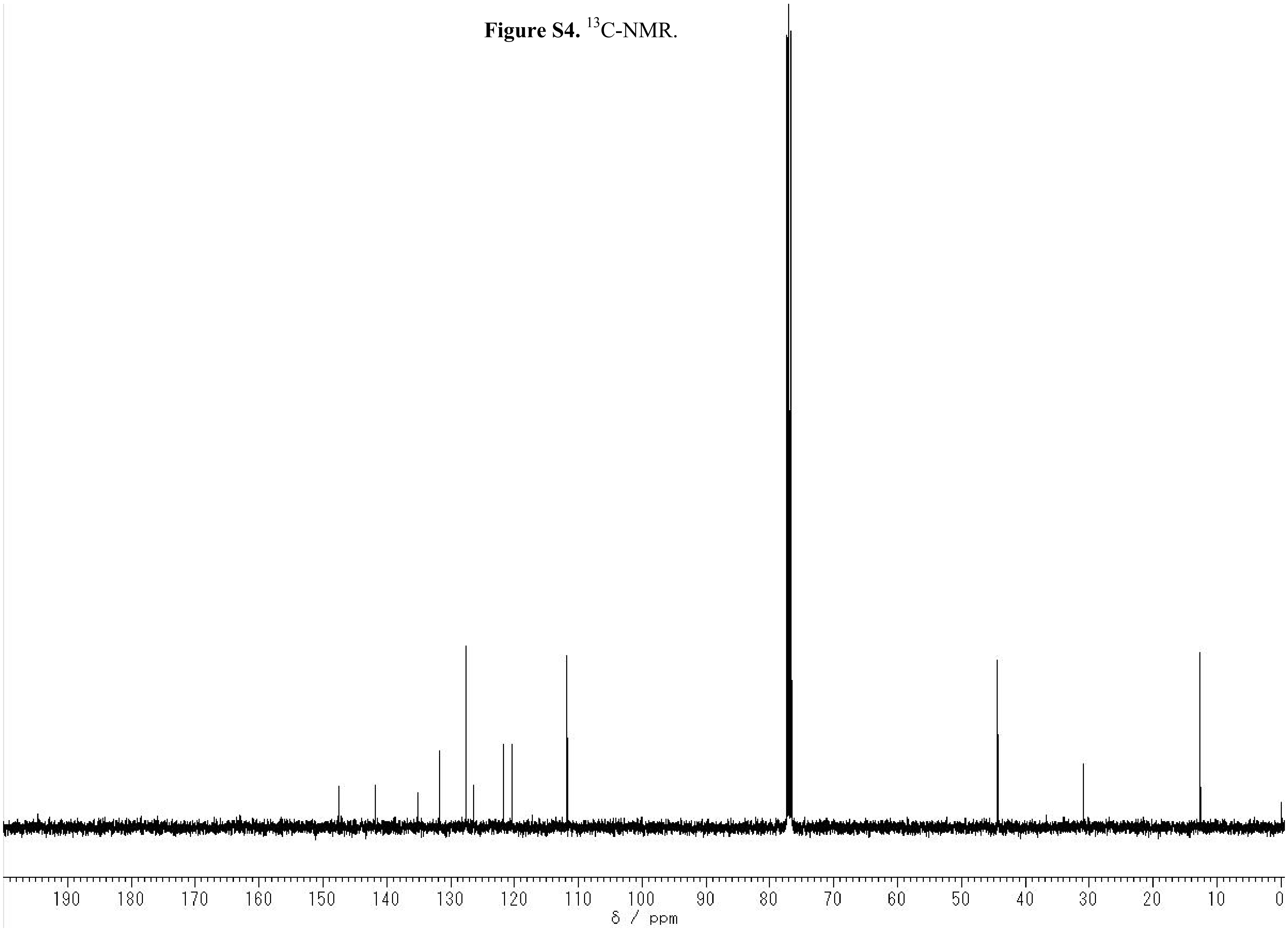

Supplement: Supplementary file 1 [file molecules-17-04452-s001.pdf]
